# Social Associative Learning and Trust Formation Across Adulthood

**DOI:** 10.1093/geroni/igaa057.1834

**Published:** 2020-12-16

**Authors:** Kendra Seaman, Alexander Christensen, Katherine Senn, Jessica Cooper, Brittany Cassidy

**Affiliations:** 1 The University of Texas at Dallas, Dallas, Texas, United States; 2 University of North Carolina - Greensboro, Greensboro, North Carolina, United States; 3 Emory University, Atlanta, Georgia, United States

## Abstract

Trust is a key component of social interaction. Older adults, however, often exhibit excessive trust relative to younger adults. One explanation is that older adults may learn to trust differently than younger adults. Here, we report a study examining how younger (N=36) and older adults (N=37) learn to trust over time. Participants completed a classic iterative trust game with three partners (15 trials each). Younger and older adults shared similar amounts but there were differences in how they shared that money. Compared to younger adults, older adults invested more with untrustworthy partners and less with trustworthy partners. As a group, older adults displayed less learning than younger adults and computational modeling suggests that older adults used different learning strategies. These findings suggest that older adults attend to and learn from social cues differently from younger adults. Neuroimaging results focused on reward processing will also be discussed.

